# Joint European policy on the COVID-19 risks for people with mental disorders: An umbrella review and evidence- and consensus-based recommendations for mental and public health

**DOI:** 10.1192/j.eurpsy.2022.2307

**Published:** 2022-08-11

**Authors:** Benedetta Vai, Mario Gennaro Mazza, Casanova Dias Marisa, Julian Beezhold, Hilkka Kärkkäinen, John Saunders, Jerzy Samochowiec, Francesco Benedetti, Marion Leboyer, Paolo Fusar-Poli, Livia De Picker

**Affiliations:** 1 Psychiatry & Clinical Psychology, Division of Neuroscience, IRCCS San Raffaele Scientific Institute, Milan, Italy; 2 University Vita-Salute San Raffaele, Milan, Italy; 3 Section of Women’s Mental Health, Institute of Psychiatry, Psychology and Neuroscience, King’s College London, London, UK; 4 Department of Psychological Medicine and Clinical Neurosciences, School of Medicine, Cardiff University, Cardiff, UK; 5 Norwich Medical School, University of East Anglia, Norwich, United Kingdom; 6 Hellesdon Hospital, Norfolk and Suffolk NHS Foundation Trust, Norwich, United Kingdom; 7 President of GAMIAN-Europe, Ixelles, Belgium; 8 Executive Director EUFAMI, Leuven, Belgium; 9 Department of Psychiatry, Pomeranian Medical University, Szczecin, Poland; 10 Université Paris Est Créteil, INSERM U955, Laboratoire Neuro-Psychiatrie Translationnelle, Fondation FondaMental, Creteil, France; 11 AP-HP, Hôpital Henri Mondor, Departement Medico-Universitaire de Psychiatrie et d’Addictologie (DMU IMPACT), Federation Hospitalo-Universitaire de Médecine de Precision (FHU ADAPT), Paris, France; 12 Department of Psychosis Studies, Institute of Psychiatry, Psychology, and Neuroscience, King’s College London, London, United Kingdom; 13 Department of Brain and Behavioral Sciences, University of Pavia, Pavia, Italy; 14 University Psychiatric Hospital Campus Duffel, Duffel, Belgium; 15 Collaborative Antwerp Psychiatric Research Institute, University of Antwerp, Antwerp, Belgium

**Keywords:** COVID-19, mental health, umbrella review, recommendations, psychiatry

## Abstract

As COVID-19 becomes endemic, identifying vulnerable population groups for severe infection outcomes and defining rapid and effective preventive and therapeutic strategies remains a public health priority. We performed an umbrella review, including comprehensive studies (meta-analyses and systematic reviews) investigating COVID-19 risk for infection, hospitalization, intensive care unit (ICU) admission, and mortality in people with psychiatric disorders, and outlined evidence- and consensus-based recommendations for overcoming potential barriers that psychiatric patients may experience in preventing and managing COVID-19, and defining optimal therapeutic options and current research priorities in psychiatry. We searched Web of Science, PubMed, and Ovid/PsycINFO databases up to 17 January 2022 for the umbrella review. We synthesized evidence, extracting when available pooled odd ratio estimates for the categories “any mental disorder” and “severe mental disorders.” The quality of each study was assessed using the AMSTAR-2 approach and ranking evidence quality. We identified four systematic review/meta-analysis combinations, one meta-analysis, and three systematic reviews, each including up to 28 original studies. Although we rated the quality of studies from moderate to low and the evidence ranged from highly suggestive to non-significant, we found consistent evidence that people with mental illness are at increased risk of COVID-19 infection, hospitalization, and most importantly mortality, but not of ICU admission. The risk and the burden of COVID-19 in people with mental disorders, in particular those with severe mental illness, can no longer be ignored but demands urgent targeted and persistent action. Twenty-two recommendations are proposed to facilitate this process.

## Introduction

According to the World Health Organization, by February 2022, 420 million people had been infected by SARS-CoV-2 and more than 5.8 million had died from COVID-19 worldwide. In Europe, confirmed cases reached 150 million with 1.8 million dead and numbers still rising. COVID-19 will most likely become endemic, identifying and protecting vulnerable populations from severe COVID-19 outcomes will remain a priority for public health. Several factors associated with mental disorders, such as a high prevalence of comorbid physical health risk conditions (e.g. smoke, obesity, diabetes, and metabolic syndrome), potential reduced access to appropriate health care, as well as immunological disturbances, suggest that psychiatric patients, in particular those with severe mental disorders, such as psychotic and bipolar disorders, represent vulnerable populations for severe COVID-19 [1–3]. During the past year, empirical evidence around the world showed the risk of COVID-19 infection, hospitalization, intensive care unit (ICU) admission, and mortality in people with pre-existing psychiatric disorders compared to people without mental disorders. The available scientific literature allowed different research groups to conduct independent systematic reviews and meta-analyses summarizing the COVID-19 risks for people with mental disorders. This paper aims to synthesize the available evidence in an umbrella review of COVID-19 risk for psychiatric patients, and provide recommendations to protect people with mental disorders and prevent avoidable deaths and healthcare complications resulting from COVID-19 infection. Our guidelines were developed based on the available evidence and on consensus between the five major psychiatric associations in Europe: European Psychiatric Association (EPA), European College of Neuropsychopharmacology (ECNP), European Federation of Associations of Families of People with Mental Illness (EUFAMI), Global Alliance of Mental Illness Advocacy Networks-Europe (GAMIAN), and European Union of Medical Specialists, Section of Psychiatry (UEMS-Psychiatry). Our recommendations are relevant to policymakers, health care professionals, and researchers in the areas of mental and public health.

## Umbrella review

### Methodology

We performed an umbrella review exploring the risk of COVID-19 infection, hospitalization, ICU admission, and mortality in people affected by pre-existing mental disorders compared to those without mental disorders, according to the Preferred Reporting Items for Systematic reviews and Meta-Analyses (PRISMA 2020, eMethod 1) [4].

We conducted an independent and systematic multi-step search of all eligible articles published up to 17 January 2022 on Web of Science (Clarivate Analytics), PubMed, and Ovid/PsycINFO databases (eTable 1 for search term, Supplementary Material). After the removal of duplicates, two authors independently completed the preliminary screening (BV and MGM) based on titles, abstracts, and full text according to the eligibility criteria. Inclusion criteria were (a) meta-analysis and systematic reviews exploring the risk of COVID-19 infection, hospitalization, ICU admission, and mortality in people affected by pre-existing mental disorders compared to those without mental disorder from original studies, and (b) written in English. Exclusion criteria were (a) studies not including people with pre-existing mental disorders or a control group without mental disorders; (b) studies not investigating associations between mental disorders and COVID-19 outcomes based on original research; (c) original studies, clinical case reports, abstracts, conference proceedings, preprints, or comprehensive studies that did not undergo a peer-review process; and (d) duplicate publications. Two authors (BV and MGM) independently extracted the data (sample size, pooled odds ratio, outcomes of interest, data for severe mental disorders, and list of subgroup analyses), and summarized these in tables. BV and MGM also assessed the methodological quality of each paper using the AMSTAR-2 (A Measurement Tool to Assess Systematic Reviews) [5] and stratified the strength of evidence [6] (eMethod 2, Supplementary Material).

### Results

**Figure 1.**
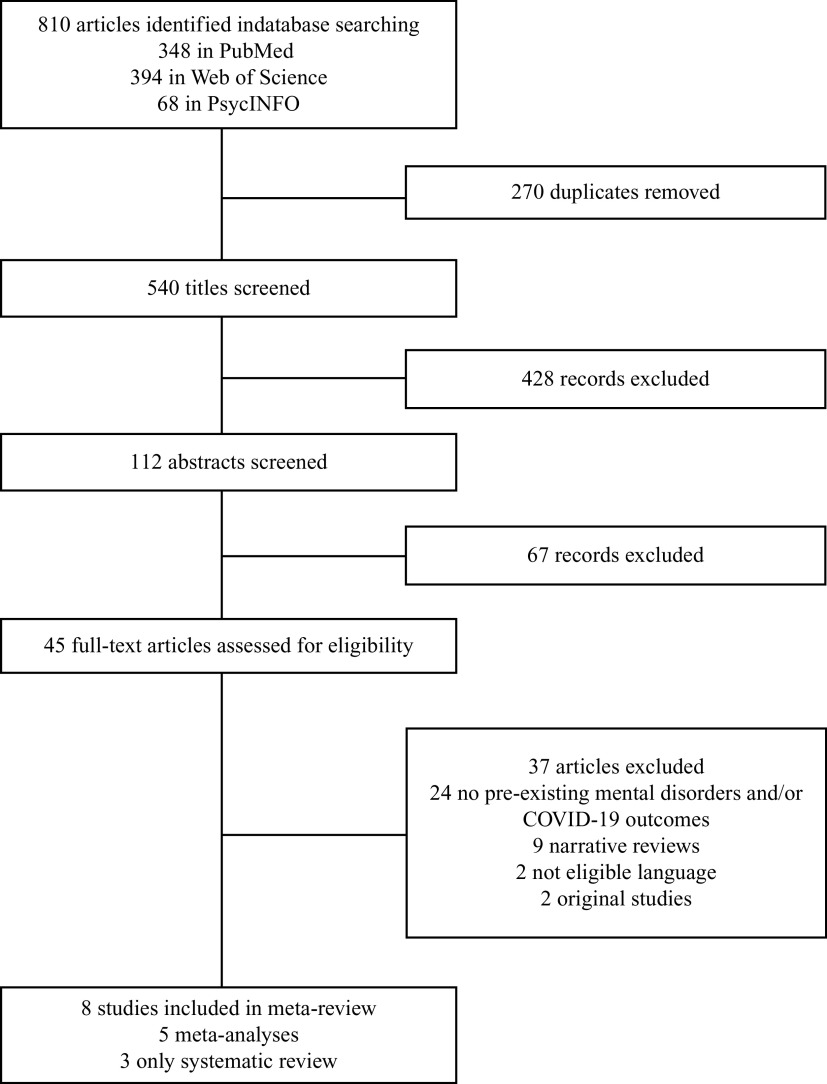
PRISMA flow chart of included studies

The literature search identified 540 studies, eight of which met the inclusion criteria (Figure 1, Tables 1 and 2) [7–14], four were combined systematic review/meta-analyses [7, 8, 10, 11], one was a meta-analysis [9], and three were systematic reviews [12–14] (eResults 1, Supplementary Material). Among these, five explored COVID-19 risk of infection [7, 8, 12–14], one explored the risk of hospitalization [11], five explored the risk of ICU admission or severe outcomes (e.g., mechanical ventilatory support, oxygen therapy, cardiopulmonary resuscitation) [7–11], and six explored the risk of mortality [7–12]. Following AMSTAR2 guidelines, two comprehensive studies were rated as moderate quality [7, 11], three as low quality [8–10], and three as critically low quality [12–14] (eResults 1, Supplementary Material). Evidence for mortality risk was found to be from suggestive to highly suggestive; severe outcomes and hospitalization risk from highly suggestive to not significant; and infection risk as not significant or weak; indicating that the evidence for increased mortality is the most robust (Table 1).

**Table 1.** Overview of meta-analyses on COVID-19 severe outcomes in any mental disorders.

						Pooled odds ratios for any mental disorder compared to people without mental disorders		Pooled odds ratios for severe mental disorders compared to people without severe mental disorders^b^			
Meta-analysis (search updated to)	No. original studies	Total sample size	Mental disorders sample size	Primary COVID-19 outcomes	Class of evidence ^a^	Crude Odds Ratio (95%CIs, I^2^)	Adjusted Odds Ratio^c^ (95%CIs, I^2^)	Crude Odds Ratio (95%CIs, I^2^)	Adjusted Odd Ratio^c^ (95%CIs, I^2^)	Additional subgroup analyses	AMSTAR 2 quality
Ceban et al. (1 Feb 2021) [8]	14	65,514,469	NA	Infection	NS	NA	NA	MD: 0·91 (0·58–1·41, 100%)	MD: 1·50 (0·75–2·99, 100%)	Depression, study design	Low
	7	26,554,397	NA	Hospitalization	IV	NA	NA	MD: 1·38 (1·17–1·63, 80%)	MD: 1·27 (1·09–1·47, 97%)		
	7	83,240	NA	Severe events	NS	NA	NA	MD: 0·93 (0·85–1·03, 0%)	MD: 0·99 (0·80–1·24)		
	12	25,808,660	NA	Mortality	II	NA	NA	MD: 1·44 (1·23–1·68, 30%)	MD: 1·57 (1·26–1·95, 78%)		
Fond et al. (22 Feb 2021) [10]	16	NA	19,086	Mortality	III	1·75 (1·40–2·20, 26%)	1·38 (1·15–1·65, 0%)	2·26 (1·18–4·31, 56%)	1·67 (1·02–2·73, 27%)	Severe mental disorders	Low
Liu et al. (7 Jul 2021) [7]	18	72,464,308	3,325,988	Infection	IV	1·42(1·15–1·76, 95%)	1·71(1·09–2·69, 100%)	MD: 2·02(1·08–3·76, 100%);^d^ SZ: 1·72(0·62–4·77, 100%)^d^	NA	Type of disorders (mental or neurological) diagnostic category, temporal relationship of exposure and COVID-19 infection (pre-existing vs sequela), sex ratio, mean age, income level of regions	Moderate
	19	25,767,005	3,045,593	Severity	II	1·40(1·25–1·57, 80%)^d^	NA	MD:1·34(1·08–1·67, 66%);^d^ SZ: 1·22(0·70–2·13, 88%)^d^	NA		
	28	34,168,377	3,211,426	Mortality	III	1·47(1·26–1·72, 93%)^d^	NA	MD: 1·36 (1·15–1·61, 81%);^d^ SZ: 2·28 (1·40–3·73, 64%)^d^	NA		
Toubasi et al. (15 Feb 2021) [9]	16	634,338	68,023	Severity and Mortality	III	1·76 (1·29–2·41, 94%)^d^	1·52 (1·20–1·93, 63%)	NA	NA	Countries	Low
Vai et al. (5 Mar 2021) [11]	23	1,469,731	43,938	Mortality	II	2·00 (1·58–2·54, 93%)	1·31 (1·13 1·52, 39%)	2·21 (1·63–2·99, 82%)	1·55 (1·30–1·85, 29%)	Diagnostic category; Psychopharmacological drug class; severe mental disorders; baseline COVID-19 treatment setting; countries	Moderate
				ICU admission	IV	1·77 (1·09–2·89, 93%)	1·33 (0·87–2·04, 90%)	1·65 (0·85–3·21, 90%)	1·08 (0·65–1·79, 69%)	Diagnostic category; severe mental disorders; baseline COVID-19 treatment setting	
				Hospitalization	II	2·24 (1·70–2·94, 89%)	1·77 (1·29–2·42, 91%)	2·48 (1·66–3·69, 55%)	1·37 (0·79–2·38, 37%)	Diagnostic category; severe mental disorders	

Abbreviations: CI, confidence intervals; ICU, intensive care unit; MD, mood disorders; NA, not available; SZ, schizophrenia.

a Meta-analytic evidence are stratified from class I to class IV and to Not significant Evidence (NS); representing respectively convincing evidence, highly suggestive evidence, suggestive evidence and weak evidence.

b Severe mental disorders included in Ref. [10] schizophrenia spectrum disorders and/or bipolar disorders, in Ref. [11] psychotic and mood disorders.

c Pooled OR adjusted in the original studies for possible confounders (e.g., age, sex, smoking, obesity, socioeconomic status, race or ethnicity, and psychical comorbidities).

d Non-fully adjusted model, including Odds Ratios from all the original studies, either crude, or adjusted.

**Table 2.** Overview of systematic reviews on COVID-19 outcomes in any mental disorders.

Systematic reviews (search updated to)	N. original studies including psychiatric disorders	Primary COVID-19 Outcomes	Conclusion	AMSTAR 2 Quality
Fornaro et al. (24 Apr 2021) [14]	1	Infection	“The present scoping review confirmed the clinical suspicions about the overall vulnerability of people with a primary diagnosis of BD compared to the general population”.	Critically low
Karaoulanis and Christodoulou (21 Mar 2021) [12]	4	Infection	“People with schizophrenia spectrum disorders constitute a vulnerable group to COVID-19. Specifically, they suffer higher rates of infection and are more likely to die from COVID-19”.	Critically low
	5	Mortality		
Murthy and Narasimha (25 Nov 2020) [13]	2	Infection	“Multiple studies suggest alcohol increases the risk of COVID-19 infection”.	Critically low

#### COVID-19 infection risk

Liu and colleagues found that any pre-existing mental disorders were associated with an increased risk for COVID-19 infection compared to people without mental disorders. The effect was confirmed even when adjusting for possible confounders (e.g., physical comorbidities, age, and smoking) [7]. When investigating risk by specific diagnostic group, meta-analysis results showed pre-existing mood [7, 8] and anxiety disorders [7] were associated with higher COVID-19 susceptibility. Systematic review also suggested higher rates of infection among people with schizophrenic [12], bipolar disorder [14], and alcohol use disorders [13].

*Summary:* Comprehensive studies consistently suggest a higher risk of COVID-19 infection for people with mental disorders compared to those without them, especially with schizophrenia, mood, anxiety, and alcohol-related disorders.

#### COVID-19 hospitalization and ICU admission risk

Specific meta-analysis estimates for hospitalization and ICU admission risks are available in three meta-analyses (Table 1): Liu et al. showed a higher risk for severe COVID-19, combining hospitalization and ICU admission, in people with mental disorders, a higher risk was specifically confirmed for mood disorders, attention deficit hyperactivity disorder and sleep disturbances, but not for schizophrenia [7]; Vai et al. found that people with psychiatric disorders, but not those with psychotic disorders, had a higher risk of hospitalization, whereas no significant differences emerged for ICU admission [11]; Ceban et al. confirmed a higher risk of hospitalization for mood disorders, however, in line with Vai et al.’s results, the risk of severe events, including ICU admission or respiratory supportive therapies, was no higher than for people without mood disorders [8].

*Summary:* Comprehensive studies suggest a higher risk of COVID-19 hospitalization for people affected by psychiatric disorders, except for psychotic disorders. ICU admission risk appears no higher for patients affected by a psychiatric disorder. This result is also replicated when specifically considering only psychotic and mood disorders.

#### COVID-19 mortality risk

Meta-analysis results and systematic review findings consistently showed higher mortality risk in psychiatric patients compared to people without mental disorders (Tables 1 and 2) [7–12]. Mortality risk was doubled for severe mental disorders (psychotic and mood disorders) [7, 10, 11], who had higher mortality estimates compared to people with other mental disorders [11]. In all the meta-analyses, the findings were confirmed in models adjusted for confounders, which mainly include age, sex, smoking, and physical comorbidity (e.g., cardiovascular and metabolic disorders, kidney or pulmonary disorders) [7–11]. Importantly, stratifying the risk by diagnostic group [7, 8, 11], results confirmed a higher risk of mortality for psychotic [7, 11, 12], mood [7, 8, 11], and substance use disorders [11], in intellectual disabilities and developmental disorders [11], but not in sleep disturbances [7], anxiety [7, 11], and stress-related disorders [7]. Higher mortality risk for schizophrenia was found in a systematic review [12]. Furthermore, Vai et al. stratified risks for psychopharmacological drug class, showing a higher mortality risk in patients treated with antipsychotics and anxiolytics [11]. However, comprehensive studies do not yet allow for differentiating the association with psychotropic drugs from that of the underlying psychiatric condition, nor for differentiating between specific pharmacological compounds. Notably, Vai et al. explored risk considering the baseline treatment setting for COVID-19, finding no evidence of increased in-hospital mortality or ICU admission. Mortality and ICU admission risks were only increased among psychiatric patients not admitted to the hospital for COVID-19 [11]. Finally, three meta-analyses stratified risk between countries: Vai et al. found the lowest mortality risks for mental disorders in Europe and the USA [11], Liu et al. found lower severe COVID-19 rates in high-income regions compared to low-income, but no significant differences for risk of mortality [7], whereas Toubasi et al. found significantly increased COVID-19 severity and mortality risk for psychiatric patients in South Korea and the USA, but not in the UK [9].

*Summary*: Comprehensive studies suggest increased COVID-19 mortality for people with mental disorders – in particular, for psychotic and mood disorders.

### Summary of the umbrella review

Our umbrella review consistently showed a higher risk of COVID-19 infection and hospitalization for people with pre-existing mental disorders compared to those without them that were not accompanied by a higher ICU admission. We also confirmed a higher mortality risk for psychiatric patients, in particular for psychotic and mood disorders. Notably, patients with psychotic disorders did not show a higher risk of hospitalization or ICU admission risk, suggesting they may be a particularly vulnerable population who also face multiple access to care barriers.

Furthermore, differences in mortality risk were detected between countries. Considering also that the higher risk of mortality remained significant even when controlling for physical comorbidities, and that mortality and ICU admission risks were only higher among psychiatric patients not admitted to the hospital for COVID-19 treatment [11], a higher mortality risk may reflect poor access to healthcare, especially for people with severe mental disorders, possibly associated to social isolation, stigma, discrimination, or scarce allocation of medical resources during COVID-19 surges [15, 16]. As limitations, most of the comprehensive studies identified were rated as low or critically low quality, with consistent evidence only for increased COVID-19 mortality, also including several retrospective and cross-sectional original studies. Furthermore, the original data, on which the comprehensive evidence was built, were published up to summer 2021 (Tables 1 and 2), prior to the emergence of the currently dominant Omicron variant. However, emerging original studies are confirming a higher SARS-CoV-2 infection susceptibility [17, 18], hospitalization [19], and mortality [18–21] for psychiatric patients. Still, it is clear that the risk and burden of COVID-19 in mental disorders, and particularly in severe mental disorders, can no longer be ignored and now demands targeted and persistent action from all stakeholders.

## Recommendations

Following our results, suggesting higher COVID-19 risks for psychiatric patients, we propose counteracting recommendations focus on (a) addressing potential disparities that may prevent psychiatric patients from accessing COVID-19 prevention and treatment strategies; (b) defining possible optimal therapeutic options for psychiatric patients, based on the emerging and preliminary findings, and (c) defining research priorities (Box 1). Our recommendations are informed by the recent literature, examples from policies across Europe, including the findings from two online surveys conducted among representatives of national psychiatric associations (EPA Council and UEMS-Psychiatry, eResults 3) and discussions with the main European psychiatry stakeholders representing patients, families, and the scientific community: GAMIAN, EUFAMI, EPA, ECNP, and UEMS.

Box 1. Recommendations

1. Access to COVID-19 vaccination and testing
  1. To remove potential social, physical, and economic barriers to vaccination and testing;
  2. To prioritize access for people with mental disorders to vaccination and testing;
  3. To engage and support caregivers in helping family members access vaccination and other preventive measures.
2. Access to COVID-19 treatment and hospitalization
  1. To remove potential social, physical, and economic barriers to access healthcare facilities providing COVID-19 treatment;
  2. To provide clear national triage guidelines, well defined for each specific clinical setting;
  3. To increase health care resources (e.g. Intensive Care Unit beds, oxygen therapies or ventilation, etc.);
  4. To improve communication within and across mental health and physical health care services.
3. Access to psychiatric care
  1. To remove potential social, physical, and economic barriers, including stigma, in accessing psychiatric care;
  2. To promote remote and digital consultations, and offer the support needed to access those, when necessary;
  3. To prioritize face to face appointments, when necessary;
  4. To assist and guide caregivers in best supporting family members.
4. Psychopharmacological treatments
  1. To follow current clinical guidelines and to remain updated with emerging literature;
  2. To carefully monitor infected patients for worsening COVID-19 symptoms; including respiratory depression, cardiovascular and thromboembolic risk possibly related to psychopharmacological compounds, to ensure patient safety;
  3. To carefully monitor drug interactions between psychotropic and COVID-19 medications
5. Inpatient, community, and collective living
  1. To support COVID-19 testing, infection control, and quarantine measures to reduce the risk of outbreaks;
  2. To support safe and regular contact for patients with their families and friends,
  3. To support those living in the community to maintain their mental health and well-being.
  4. Family members involved in caring support should have access to professional support and guidance to ensure that they can continue their role as carers to their loved ones.
6. Research priorities during the pandemic
  1. To identify social, psychological, and biological factors underlying COVID-19 suscetibility and severity in people with mental disorders;
  2. To explore how risks may change for vaccinated or previously infected people, and in different clinical psychiatric settings (e.g. inpatient, outpatient, or community settings), or pandemic phases;
  3. To identify factors contributing to vaccine uptake and vaccine hesitancy in people with mental disorders;
  4. To perform studies on COVID-19 drug safety and effectiveness in people with pre-existent mental disorders and taking psychotropic medications.

### Addressing inequalities

#### Vaccine uptake and COVID-19 testing

Parallel to a higher risk of COVID-19 infection, psychiatric patients, particularly those with severe mental disorders, may experience barriers to access immunization or testing related to lack of knowledge, awareness, or trust, difficulties applying the law, as well as practical problems related to accessibility and costs [19, 22, 23], possibly resulting in a lower vaccination rate. Policy makers are directly called upon to promote and facilitate vaccination and COVID-19 testing for psychiatric patients. Potential barriers to vaccination should be removed: mental health services can help patients to access vaccination hubs, or directly administer vaccines (previous findings showed that targeted vaccination programs offered by mental healthcare services resulted in vaccine uptake similar to the general population [24, 25]), and respecting ethics and law on human rights when making decision for people with and without capacity. COVID-19 testing should be made available and easily accessible, preferably free, in multiple locations accessible by public transport, and without advance or online registration, as well as in mental health departments and at home. In case of limited vaccination or testing capacity, psychiatric patients should be prioritized considering their increased risk of developing severe COVID-19, according to WHO [26], European Commission guidelines [27], and EPA council report (eResults 3.1, Supplementary Material). Furthermore, caregivers are a key support system for patients with mental disorders, who can provide invaluable help with accessing vaccination or other preventive and treatment measures. Policies focused on targeted communications with families and caretakers in the community may help improve the level of access to appropriate prevention and treatment. In general, the inclusion of mental healthcare representatives should be considered in all strategic planning of COVID-related public health measures.

#### Access to COVID-19 treatment and hospitalization

Psychiatric patients may experience difficulty accessing appropriate medical care due to social isolation, stigma, discrimination, physical, or economic barriers or even, when hospitalized, they may be subjected to “healthcare rationing” due to the scarce allocation of resources and/or to a misinterpretation of ICU triage protocols and guidelines [28]. Barriers and inequalities for access to COVID-19 healthcare facilities may contribute to our umbrella review findings: a higher risk of COVID-19 mortality, in combination with a non-increased risk of hospitalization and ICU admission in psychiatric patients, especially those with severe mental disorders or intellectual disabilities [28]. These results may also express a more rapid and severe disease progression in psychiatric patients, due for example to the high somatic comorbidities (e.g., cardiometabolic disease) [1–3], which may prevent patients from reaching timely healthcare services. Nevertheless, policies ensuring equal access to healthcare, including a clear definition of national triage guidelines, as well as potentiation of available resources are mandatory. Furthermore, each healthcare facility should adapt general guidelines to local circumstances to ensure the best clinical practice in caring for psychiatric patients with COVID-19, in collaboration with local ethics committees and psychiatric services. Policies facilitating communication and cohesion within and across mental health, primary, and hospital care services are highly welcomed: adoption of rapid, flexible, efficient, and even remote methods, as well as digital medicine can be valuable instruments for reaching and monitoring psychiatric patients, even in quarantine. Early referral for hospitalization or ICU admission should be facilitated in case of deterioration with COVID-19 symptoms, also possibly counteracting rapid disease progressions.

### Psychiatric treatment

#### Access to psychiatric care during the pandemic

Psychiatric patients may experience barriers and difficulties also in reaching psychiatric care. During the first waves of the pandemic, data worldwide demonstrated a reduction in access to psychiatric facilities, even hospitalization, as shown by the UEMS-Psychiatry evaluation (eResults 3.2, Supplementary Material) that also related to perceived stigma and discriminant behaviors [29–33]. Reduced access to psychiatric care may increase the risk of relapses and worsening of psychopathology, identifying per se a further risk factor for COVID-19 infection and worse outcomes, related to a lower compliance to protective measures and reduced ability to self-monitoring or self-caring of COVID-19 symptoms or their ability to reach adequate medical care, when necessary. Furthermore, psychiatric facilities and the involved health-care professionals may directly help patients in reaching COVID-19 preventive facilities (vaccination and testing), and in eventually monitoring the COVID-19 disease course facilitating a rapid referral to hospitalization.

Moreover, disruption of mental health services, paralleled by the social isolation experienced during the pandemic, may have deeply burdened not only patients, but also their families, that can provide important support to patients when eventually infected. Policies reducing stigma and increasing awareness in the general population, and generally facilitating access to mental health care facilities, including remote and digital psychiatric, psychotherapies or psychological support consultations should be prioritized. Challenges related to inequalities in internet or care access, abilities using technology, privacy, and economic issues should be assessed, also considering the probable increase in demand for psychiatric care [34–36]. Professionals should have access to a continuous medical education (CME) to quickly become equipped with new knowledge and technology. Caregivers should have access to professional assistance and guidance to best support their family members.

#### Psychopharmacological treatment

*Antidepressants.* Meta-analysis evidence suggested that while mood disorders are associated with a higher risk of COVID-19 severe outcomes [7, 8, 11], pre-existing antidepressant treatment was not associated with a worse prognosis after adjusting for age, sex, and other covariates [11]. Interestingly, some current evidence supports the potential role of antidepressants as effective repurposed early treatment options for preventing clinical deterioration in SARS-CoV-2 infected patients [37]. Anti-inflammatory, immune-modulatory, and antiviral properties of conventional antidepressants can mediate this protective effect for severe COVID-19 outcomes [38]. Despite all our recommendations for psychopharmacological treatments should be considered with caution, considering the paucity of studies, antidepressants appear as possible safe compounds, while deeply monitoring drug-to-drug interaction with COVID-19 medication.

*Antipsychotics.* The effect of antipsychotic treatment on COVID-19 severe outcome is still a matter of debate [39]. While meta-analysis evidence suggests an association between exposure to antipsychotics and COVID-19 mortality [11], single studies did not confirm this association [40, 41]. The inconsistency between studies could be explained by heterogeneity among different antipsychotic classes and treatment adherence. Specific antipsychotic classes are known to differentially precipitate cardiovascular and thromboembolic risk and cause drug-to-drug interaction with COVID-19 medication [42–44]. Moreover, current evidence suggests that aripiprazole could reduce the COVID-19 inflammatory response [45]. Notably, when dealing with antipsychotics, poor treatment adherence is associated with an increased risk of psychiatric relapse and decreased adherence to healthcare recommendations. A study, where an inpatient setting ensured antipsychotic drug adherence, reported that patients were less likely to contract COVID-19 and had better outcomes following infection than the general population [41]. In the absence of clear evidence about the detrimental role of antipsychotics, we recommend clinicians to follow current clinical guidelines including close clinical monitoring of patients to achieve good treatment adherence.

*Mood stabilizers.* One study showed that lithium improves inflammatory activity and immune response in six patients with severe SARS-CoV-2 infection [46]. Furthermore, therapeutic lithium levels were also consistently associated with lower risks of COVID-19 infection compared to patients using other mood stabilizers or subtherapeutic doses [47]. Based on available evidence, mood stabilizers are relatively safe in the case of COVID-19. Treatment should be continued in the context of strict monitoring of serum levels, hydration, renal, and liver functions and considering drug interactions, side effects, and the narrow therapeutic range of this psychopharmacological class [42, 48].

*Benzodiazepines and other sedatives.* Meta-analysis evidence suggested that exposure to anxiolytics was associated with a higher risk of severe COVID-19 outcomes [11]. Benzodiazepine and anxiolytics can be associated with the risk of respiratory distress, which is higher for highly sedative agents, at higher doses, and when prescribed in patients with pre-existing respiratory impairment [49]. Clinicians prescribing benzodiazepines and anxiolytics should be aware of the risk of respiratory depression in the context of COVID-19 and the importance of careful monitoring of their patients.

#### Inpatient treatment and collective living

Psychiatric hospitals and inpatient or collective settings are a high-risk environment for major outbreaks [50–53]. COVID-19 rapid or PCR testing and/or quarantine before or upon admission may help in reducing outbreaks. COVID-19 testing and isolation should be used as preventive measures in case of high-risk contacts or onset of suspect symptomatology, and specific protocols for confirmed cases should be designed. Evidence from previous outbreaks has indicated that measures to prevent transmission from staff to patients are particularly important. Compliance with containment measures such as social distancing, disinfection, and mask wearing should be advised; where this is difficult, possible compartmentalization of patients and health-care professionals can be considered. Adequate resources (such as PCR testing, masks, and disinfectants) should be made available to mental healthcare facilities [54]. In general, lockdowns should be avoided: psychiatric patients, and their families, may benefit from regular contact with the outside world, whereas prolonged home confinement may increase the levels of distress and social isolation, also worsening psychiatric symptomatology [55–58]. Regarding potential future pandemics, a solid effort should ensure that people with mental health disorders, and their families, receive sufficient support to maintain optimal well-being.

### Research priorities during the pandemic

#### Factors underlying COVID-19 susceptibility and severity in people with mental disorders

Several factors, including social, psychological, and medical ones, were hypothesized to contribute to a higher risk of infection and severe outcomes in psychiatric patients. Although it is also reasonable to assume that multiple factors, including psychopathology itself, or socioeconomic factors (e.g., collective living, residential instability), and on the other hand, potential bias in a higher or lower rate of testing for psychiatric patients [59, 60] may affect the detected estimates and the risk to be infected, and barriers to treatment, isolation, stigma, lifestyle, comorbidities, interaction with psychopharmacological compounds or immuno-inflammatory dysregulations, may contribute to severe COVID-19 outcomes, no clear explanation has yet emerged from research [11]. Clarifying the underlying causes for increased COVID-19 risk of infection and mortality of psychiatric patients will provide new important clinical insights. Research also needs to explore how risks may change over the different phases of the pandemic, stratifying for vaccinated or previously infected patients, who should be protected from COVID-19 infection, and maybe severe outcomes, and for different clinical psychiatric settings (e.g., in inpatient, outpatient, or community samples) [61]. Furthermore, knowing that mental disorders, in particular severe ones, are characterized by abnormalities in the immunoinflammatory system [62], the effect of vaccine inoculation or infection should be studied.

#### Factors determining vaccine uptake in people with mental disorders

Despite current literature suggesting that psychiatric patients may experience barriers to reach immunization, further literature is required. In particular, future research should be focused on evaluating the consistency, strongness, and eventual source heterogeneity in the detected effects, and in identifying factors contributing to vaccine uptake. In a population where comorbidity with physical health disorders and socio-economic factors may additively lead to a higher COVID-19 vulnerability and inequality, as in psychiatric disorders, it is of crucial relevance [63]. Different barriers may contribute to low vaccine uptake in mental disorders, including low vaccine education, costs, and difficulties in accessing healthcare facilities [23], as well as vaccine hesitancy: “a delay in acceptance or refusal of vaccines despite availability of vaccination services” [64]. Vaccine hesitancy is influenced by factors including confidence (trust in the vaccine or provider), complacency (seeing the need or value of vaccination), and convenience (easy, convenient access to vaccination) [65, 66], which in turn may be affected by specific sociodemographic (e.g., age, sex, education, annual income) [67–70] and psychopathological dimensions (e.g., anxiety, panic attacks, agoraphobia, delusions, paranoia, depression) [71]. As previously shown, vaccine uptake in psychiatric patients can be increased by targeted prevention programs [23]. Defining determinants of COVID-19 vaccine uptake will help tailor specific policies and health communications [72].

#### Pharmacological treatment options for COVID-19 for people with mental disorders

Several treatment options have been authorized by the EMA and FDA including antivirals (remdesivir and ritonavir), cytokine blocking agents (anakinra and tocilizumab), and monoclonal antibodies (regdanvimab, casirivimab/imdevimab, and sotrovimab). Moreover, corticosteroids, antibiotics, chloroquine, eparine, paracetamol, and non-steroidal anti-inflammatory have been regularly used [73]. Due to the high incidence of somatic comorbidities in psychiatric disorders (e.g., cardiovascular and metabolic disorders, coagulopathies, and immunological disorders), and possible drug interactions that can result in either reduced drug tolerance, efficacy, or safety [43, 44, 74], caution should be paid when prescribing specific classes of drugs in psychiatric patients. Studies on drug safety and effectiveness in people with pre-existent mental disorders and using psychopharmacological treatments are required.

## Conclusion

Currently, the COVID-19 pandemic continues with new variants of the virus, and steep increases in positive cases. To ward off the next wave of the pandemic and to accommodate the anticipated endemicity of COVID-19, many governments are now defining new policies and strategies, including rapid or repeated deployment of booster vaccinations to the adult population. This presents a valuable opportunity to adjust and optimize existing approaches toward people with mental disorders and their involved family members. Based on our review of the literature and engagement with stakeholders, we suggest recommendations for overcoming potential inequalities and barriers that may prevent psychiatric patients and their families from accessing timely to preventive and therapeutic measures for COVID-19; recommendations for accessing optimal psychiatric care; and research priorities. These recommendations provide a foundation for evidence-based guideline development for different stakeholders. A rapid and constant update of the proposed recommendations is however necessary considering the possible evolving of the COVID-19 pandemic, and counteractive measures.

## Data Availability

Data sharing not applicable – no new data generated.

## Supplementary Materials

To view supplementary material for this article, please visit http://doi.org/10.1192/j.eurpsy.2022.2307.
